# Frailty and physical function in chronic kidney disease: the CanFIT study

**DOI:** 10.1186/s40697-015-0067-4

**Published:** 2015-09-05

**Authors:** Simon R. Walker, Ranveer Brar, Frederick Eng, Paul Komenda, Claudio Rigatto, Bhanu Prasad, Clara J. Bohm, Leroy J. Storsley, Navdeep Tangri

**Affiliations:** Seven Oaks Hospital, 2PD-13 2300 McPhillips Street, Winnipeg, Manitoba R2V 3M3 Canada; Health Sciences Centre, Winnipeg, Manitoba Canada; University of Manitoba, Winnipeg, Manitoba Canada; Regina Qu’Appelle Health Region, Regina, Saskatchewan Canada

## Abstract

**Background:**

Frailty, a manifestation of unsuccessful aging, is highly prevalent in people with chronic kidney disease (CKD) and is associated with comorbid conditions in cross-sectional studies. Longitudinal studies investigating the progression of frailty in those with advanced non-dialysis CKD are lacking.

**Objectives:**

*Can*adian *F*railty Observation and *I*nterventions *T*rial (CanFIT). To determine the natural history, prevalence of perceived and measured frailty and its association with dialysis treatment choices and adverse outcomes in patients with advanced CKD.

**Design:**

Longitudinal observational study, designed to collect data from 600 participants over 2 years.

**Setting:**

Interprofessional non-dialysis CKD clinics at four tertiary health care centres in central Canada.

**Patients:**

People with CKD stage 4 and 5 (eGFR <30 ml/min/1.73 m^2^) who are not on dialysis at enrollment.

**Measurements:**

Multiple Frailty Definitions: Short Physical Performance Battery (SPPB), Fried Frailty Criteria, Frailty Index. Dialysis start: In-Centre Hemodialysis, Home Hemodialysis or Peritoneal Dialysis Outcomes: Death, Opt-out or Lost to follow up.

**Methods:**

We will perform physical and cognitive assessments annually. We plan to analyze the relationships between frailty, treatment choices and patient centered outcomes.

**Results:**

We have recruited 217 participants in 2 centres; of these, 56 % had reduced physical function at baseline, as defined by the SPPB. Risk of reduced physical function was 8 fold higher in those with diabetes after adjusting for age, gender, eGFR and comorbidities.

**Limitations:**

Referred population, use of SPPB as a measure of frailty, inter-operator variability in measurement of hand grip and gait speed, cross-sectional analysis of baseline data in the subset recruited to date.

**Conclusions:**

People with advanced CKD have a high burden of reduced physical function, especially those with diabetes. We will continue enrollment into the CanFIT study to further understand the clinical history of CKD and frailty in this population.

## Résumé

**Contexte:**

La fragilité, une manifestation du vieillissement malheureux, est très répandue chez les personnes atteintes d’insuffisance rénale chronique (IRC) et est associée à des conditions de comorbidité dans les études transversales. Rares sont les études longitudinales destinées à étudier la progression de la fragilité chez les personnes atteintes d’IRC avancée qui ne reçoivent pas de dialyse.

**Objectifs:**

*Can*adian *F*railty Observation and *I*nterventions *T*rial (CanFIT). Déterminer l’évolution naturelle, la prévalence de la fragilité perçue et mesurée, de même que son association avec les options de traitement à la dialyse et les effets indésirables sur les patients atteints d’IRC avancée.

**Type d’étude:**

Étude longitudinale d’observation visant à recueillir les données de 600 patients sur deux ans.

**Contexte:**

Des unités interprofessionnelles d’IRC qui ne pratiquent pas la dialyse dans quatre centres de soins tertiaires du centre du Canada.

**Participants:**

Des personnes atteintes d’IRC de stade 4 et 5 (R-EGF <30 ml/min/1,73 m^2^) qui ne recevaient pas de dialyse au moment de l’inscription.

**Mesures:**

Diverses définitions de la fragilité : le Short Physical Performance Battery (SPPB), les critères de fragilité de Fried et l’indice de fragilité. Le lieu de l’amorce de la dialyse : la dialyse en centre, la dialyse à domicile; ou les résultats de la dialyse péritonéale : le décès, le refus ou la perte de suivi.

**Méthodes:**

Nous effectuerons des examens physiques et cognitifs sur une base annuelle. Nous planifions analyser la relation entre la fragilité, le choix du traitement et les résultats axés sur le patient.

**Résultats:**

Nous avons recruté 217 participants dans 2 centres; parmi ceux-ci, 56 % présentaient d’entrée de jeu une réduction des fonctions physiques, telles que définies par le SPPB. Les risques de subir une réduction des fonctions physiques étaient 8 fois supérieurs chez les patients souffrant de diabète, après ajustement selon l’âge, le sexe, le R-EGF et les comorbidités.

**Limites de l’étude:**

La population désignée, le recours au SPPB pour mesurer la fragilité, la variabilité des intervenants dans la mesure de la vitesse de préhension et de marche, l’analyse transversale des données de référence du sous-ensemble recruté jusqu’à maintenant.

**Conclusions:**

Les personnes atteintes d’IRC avancée sont accablées d’une forte réduction de la fonction physique, et particulièrement celles qui sont atteintes de diabète. Nous poursuivrons l’inscription à l’étude de CanFIT afin d’approfondir les connaissances au sujet de l’évolution clinique de l’IRC et la fragilité des personnes atteintes.

## What was known before?

People with mild to moderate CKD are more likely to be frail than those without CKD and those who have CKD and frailty are more likely to have poor outcomes. The clinical history of frailty in people with advanced CKD (eGFR <30 ml/min/1.73 m^2^) is poorly understood.

**Fig. 1:**
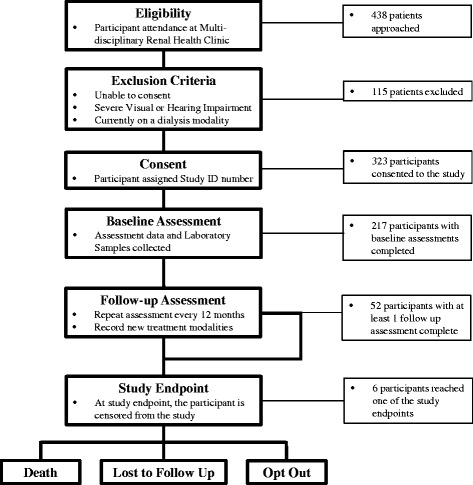
Study Participant Flow (CANFIT). Flow diagram of CanFIT (Canadian Frailty Observation and Interventions Trial) participants included in the study

## What this article adds?

We will describe the correlates of frailty in people with advanced CKD and describe its trajectory. We will identify predictors of incident frailty and of progression of frailty. We will use this knowledge better to design studies of interventions to prevent or delay poor outcomes before the onset for kidney failure.

## Background

Chronic kidney disease (CKD) affects 3 million adults in Canada and these individuals are at risk for premature death and kidney failure [1]. Although CKD affects approximately 10 % of the general population, more than one third of elderly individuals have CKD [2]. In the elderly, CKD is associated with increasing comorbid conditions, a higher risk of cardiovascular disease, and increasing levels of frailty and disability [3].

Frailty is a multidimensional syndrome, comprising physical and cognitive impairments, decreased physiological reserves and poor response to stressors [4]. Patients with CKD are at increased risk of frailty and frail CKD patients may suffer from worse outcomes. Both CKD and frailty may be surrogates for unsuccessful aging. Several definitions and approaches to the identification of frailty have been proposed. The ‘Frailty Phenotype’, as defined by Fried et al., has become widely used, especially in studies of patients with CKD. This definition of frailty comprises five domains: slowness, weakness, weight loss, low activity and fatigue [4]. Frailty is defined as the presence of significant deficits in three of the five domains and is associated with increased risk of falls, hospitalization and death [4]. In addition to the Fried criteria, poor cognition has been proposed as a domain of frailty and has also been associated with poor outcomes [5].

Other definitions of frailty have been described. The Frailty Index identifies deficits from a pre-defined list of comorbid conditions, functional deficits and laboratory variables, and defines frailty as the occurrence of a threshold number of these deficits [6]. This definition provides more flexibility by describing frailty along a continuum rather than dichotomously as in the Fried Criteria. However, the Frailty Index requires the ability to track many variables and is more difficult to apply in a clinical setting. Another method for measuring frailty is the Short Physical Performance Battery (SPPB). The SPPB involves 3 simple tests; chair stand, gait speed and balance [7]. This test is easy to administer and is readily standardized, using a scoring system of 0–12, defining normal physical function at a specific score. However, the SPPB focuses primarily on physical ability and may not capture the multidimensional nature of frailty.

Early studies of frailty and CKD have used heterogeneous definitions, and focused on patients on dialysis. More recently, studies have attempted to capture more domains of frailty and have found that the burden of frailty in individuals on dialysis and with moderate CKD (Stage 3 CKD, defined by estimated glomerular filtration rate (eGFR) 30–60 mL/min/1.73 m^2^) is exceptionally high [8, 9]. Although a link between frailty, CKD and poor outcomes has been demonstrated, most studies have used cross-sectional design and have applied different definitions of frailty (physical vs deficit based vs multi system), with limited comparisons between frailty definitions in the same population.

Furthermore, none of the studies included significant numbers of patients with CKD stages 4 and 5 (eGFR <30 ml/min/1.73 m^2^), where frailty is more common. The natural history of frailty in people with CKD stages 4 and 5 and its association with adverse outcomes is poorly understood. We are therefore conducting the CanFIT (*Can*adian *F*railty Observation and *I*nterventions *T*rial) study in order to understand the clinical history of frailty in this population and its association with worsening kidney function, adverse outcomes, and dialysis treatment modality decisions. This manuscript describes the methodology of the CanFIT study and reports on the baseline characteristics of the cohort recruited to date.

## Methods

### Objectives

We are conducting the CanFIT study in order to understand the natural history of frailty in this population, the connection between frailty, worsening kidney function and adverse outcomes, and the impact on treatment modality decisions. The objective of the CanFIT study is to determine the clinical history, prevalence of perceived and measured frailty and its association with dialysis treatment choices and adverse outcomes in patients with advanced CKD. Secondary downstream objectives are to evaluate and pilot interventions aimed at reducing frailty in patients who are at high risk for these adverse outcomes.

### Study design

This multicentre, longitudinal observation cohort study began enrolling participants in September 2012 and is designed to collect data from 600 participants over 2 years. After obtaining consent, each participant undergoes a physical assessment at their next clinic visit (3–6 months after Date of Consent). Participants will be followed up by repeating the assessment annually (every 9–15 months, depending on clinic appointment dates) until they reach a study endpoint; Death, Opting Out or Loss to Follow Up. We have developed detailed standard operating procedures which detail definitions, methods, patient flow, audit procedures, etc. New staff members are trained using these protocols. Ethics approval obtained from University of Manitoba Health Research Ethics Board on March 28, 2012 and renewed annually (Ethics Reference Number: H2012:001). Approval also obtained from St. Boniface Hospital Research Review Committee on June 20, 2013 (Reference Number: RRC/2013/1294).

### Study participants

Individuals with Stage 4 or 5 CKD, defined by eGFR >30 mL/min/1.73 m^2^, are approached to participate in the study if they attended an interprofessional non-dialysis CKD clinic at one of three sites in Winnipeg, MB (Sites: Seven Oaks General Hospital, St. Boniface General Hospital, Health Sciences Centre) or one site in Regina, SK (Kidney Health Centre at Regina General Hospital). Individuals are excluded if they are incapable of informed consent, if they are unable to speak English, or of they have been treated with a dialysis modality before their first assessment. Individuals who are blind are excluded from participating, however, visually impaired individuals were included if they retained some visual ability. Please see Fig. 1 for the flow of participants through our study.

### Data collection

Upon entry to the study, demographic information is recorded (date of birth, gender and race). At each assessment, comorbidities, case report form questions, questionnaires, physical function tests, the physician and nurse impression of frailty is recorded and chart review information is collected (comprising laboratory results, adverse events and additional comorbidities).

#### Comorbidities

Comorbidities are recorded in two groups: Self-reported and Case Summary reported. Self-reported comorbidities are assessed by recording the participant’s response to a predetermined question (e.g., ‘have you ever been diagnosed with asthma by a doctor?’). Self-reported comorbidities include: Asthma, Arthritis, Visual Impairment, Hearing Impairment, Depression, Anxiety/Panic Attacks, Malignancy, and Psychological Stress/Acute Disease. Case Summary Reported comorbidities are collected by searching for terms or synonyms on the participant’s case summary (found in their clinic chart, adjudicated by their nephrologist and reviewed by the interprofessional team on an annual basis). If the Case Summary lists the comorbidity or a synonym, the comorbidity is marked as present. Case Summary reported comorbidities include: Myocardial Infarction (MI), Prior angioplasty or stent, Prior cardiac surgery, Diabetes (Type I or II), Hypertension, Dyslipidemia, Peripheral Arterial Disease, Stroke, Cerebrovascular disease (i.e., Transient Ischemic Attack), Other Neurologic Disease (i.e., Parkinson’s, Alzheimer’s, Huntington’s or Multiple Sclerosis), Cirrhosis, Gastro-intestinal Disease, Pulmonary Hypertension, Chronic Obstructive Pulmonary Disease (COPD), Congestive Heart Failure (CHF). A free text field is also included to collect any comorbidities not captured by the included list.

#### Case report form questions

Participants are asked for responses to several questions: weight loss within 3 and 12 months, number of falls within 1, 3 and 12 months, use of mobility aids within the last year (defined as any objects used to assist the participant with self-transportation, such as a cane, walker, wheelchair, etc.), new living arrangements within the last year and hospital admission and reason within the last year (defined as 24 h or greater stay in hospital). The participant is also asked for their height (in inches or cm), due to clinic resource constraints; this is used to calculate a body mass index (BMI).

#### Questionnaires

The questionnaires administered are: Centre for Epidemiological Studies Depression Scale (CES-D, 2 Item version, adapted from Cardiovascular Health Study) [4], Geriatric Depression Scale (GDS, 5 item version) [10, 11], Montreal Cognitive Assessment (MoCA version 1.0) [12], EQ5D and Visual Analogue Scale (VAS) (Version 1.0, 2007) [13], Physical Activity Scale for the Elderly Survey (PASE) [14]. Each questionnaire is scored according to the official instructions included with the questionnaire. A participant is scored as ‘Exhausted’ if they answered ‘Occasionally’ or ‘Most or all of the time’ to either question on the CES-D. A participant is scored as ‘Depressed’ if they answered positively for 2 or more of the 5 questions for the GDS. The MoCA is scored according to MoCA Version 1 instructions. EQ5D is reported as a string of the responses grouped together (e.g., 32121) and EQ-VAS is reported as a whole number, from 1 to 100. The PASE is scored using the included scoring system and results are reported as both PASE score and Paffenbarger Physical Activity Index score (kcal/week).

#### Physical tests

Patients are asked to perform the Short Physical Performance Battery (SPPB) and Handgrip Strength tests at each assessment. The SPPB (adapted for 4 m walking distance) contains 3 balance tests (side by side, semi-tandem and tandem), chair stand test and 4 m gait speed test. Handgrip strength is measured using a Jamar Hydraulic Dynamometer (Model J00105, LaFayette Instrument Company Inc.), measured 2 times on each hand and all values recorded in kilograms. The SPPB is scored from 0 to 4 in 3 sections (see Table 1); balance tests (Side by side, semi-tandem and tandem), chair stand test and 4 m gait speed test. Ability to perform side by side and semi-tandem balance tests are scored 1 pt = ≥10s, 0pts = <10s or unable. Ability to stand in tandem stance is scored: 2pts = 10s, 1 pt = 3-10s, 0 pt = <3 s or unable. The chair stand test is scored from 0 to 4 based on pre-established time cut offs, 4 pts = ≤11.19 s, 3 pts = 11.20–13.69 s, 2 pts = 13.70–16.69 s, 1 pt = ≥16.70s, 0 pts = >60s or unable. The 4 m gait is scored from 0 to 4 based on pre-established time cut offs, 4pts = <4.82 s, 3pts = 4.82–6.20s, 2pts = 6.21–8.70, 1pts = >8.70s, 0pts = unable.

**Table 1:** Short physical performance battery scoring [7]

Test		Scoring		Total
Chair stand test	The time taken for the participant to rise from sitting in a chair 5 times is measured. The test is completed without using hands on the chair or other tools to help the participant stand.	0	Unable or >60s	4 points
		1	≥16.70s	
		2	13.70–16.69 s	
		3	11.20–13.69 s	
		4	≤11.19 s	
Balance tests	Side by Side: the participant is asked to stand with both feet side by side and the time is measured.	0	Unable or <10s	4 points
		1	≥10s	
	Semi-Tandem: the participant is asked to stand with one foot slightly more in front of the other and the time is measured.	0	Unable or <10s	
		1	≥10s	
	Tandem: the participant is asked to stand with one foot in front of the other and the time is measured	0	Unable or <3 s	
		1	3.00–9.99 s	
		2	≥10s	
4 m gait speed test	The time taken for the participant to walk 4 m is measured twice. The average time of the two trials is used to calculate score. Use of a mobility aid in the test was recorded.	0	Unable to Complete	4 points
		1	>8.70s	
		2	6.21–8.70s	
		3	4.82–6.20s	
		4	<4.82 s	

The SPPB is scored from 0 to 4 in 3 sections for a maximum score of 12 and minimum score of 0. Scores were grouped by normal physical function and frail: score ≥10 (normal physical function) and score <10 (reduced physical function as measure of frailty)

#### Chart review

After the assessment, the research coordinator obtains the laboratory values from the participants chart (using laboratory test dates on the closest date to the participant’s clinic visit), including: Hemoglobin (mg/L), Creatinine (umol/L), eGFR (mL/min/1.73 m^2^), Blood Glucose (mmol/L), Serum Calcium (mmol/L), Serum Phosphate (mmol/L), Serum Albumin (mmol/L), Alkaline Phosphatase (U/L), Aspartate Transaminase (U/L), Alanine Transaminase (ALT), Urine Albumin Creatinine Ratio, Hemoglobin A1C (%), Parathyroid Hormone (ng/mL), Low Density Lipoprotein (mmol/L), High Density Lipoprotein (mmol/L) and Triglycerides (mmol/L). Other values extracted from the participant’s chart include: Weight (kg), Systolic Blood Pressure (mmHg), Diastolic Blood Pressure (mmHg), Treatment Plan (i.e., Hemodialysis, Peritoneal Dialysis, Home Hemodialysis or no Renal Replacement Therapy) and Case Summary Comorbidities.

#### Study outcomes

Participants will be followed until death, or opting out, or loss to follow up. Participant death will be confirmed by medical records review. Date of death and cause of death will be recorded. If a participant opts out of future participation in the study, their permission is requested to use the data and laboratory samples already collected. If permission is denied, the data and laboratory samples are destroyed. If permission to use the existing data and laboratory samples is given, the data remains in the data set, but the participant is no longer followed up. A participant is defined as ‘Lost to Follow Up’ if they are no longer attending one of the clinics associated with the study and were not accessible to the research coordinators. Starting another treatment modality after the first assessment, such as peritoneal dialysis or hemodialysis is not defined as a study endpoint.

Other outcomes collected during the study include morbidity, falls, new disability and new dialysis modality. Morbidity outcomes will be collected using the Self-reported Comorbidities, Case Summary Comorbidities and self-reported hospital admissions collected during the assessment. At the 3 year point of the study, outcomes will be investigated by linking personal health identifier numbers with databases including the Canadian Institute of Health Information-Discharge Abstract Database (CIHI-DAD), Manitoba Drug Program Information Network (DPIN), Saskatchewan Pharmaceutical Information Program (PIP) and by linking to physician claims for hospitalizations. These linkages will be performed at the Manitoba Center for Health Policy. This will allow for information such as hospitalizations, pharmaceutical drug use and health outcomes to be collected. Kidney failure, defined by a need for dialysis or kidney transplant and death will be confirmed by linkage to the Manitoba Renal Program (MRP) database.

#### Plan for future analysis

Frailty will be measured using the Fried’Frailty Phenotype’, Rockwood Frailty index, physician and nurse perception, as well as the SPPB. Future analysis will involve applying these other definitions of frailty to the dataset. The Fried Frailty Criteria (a.k.a. the Frailty Phenotype) was established by Fried et al. in 2001. This method measures 5 domains of frailty; Weight Loss, Weakness, Exhaustion, Slowness and Low Activity. Standardized criteria are applied to determine whether a participant’s score is frail or not. For example, an individual is defined as frail in Weight Loss if the report more than 10 lbs of unintentional weight loss within the last 12 months. If a participant is positive for three or more of the criteria, they are classified as frail. In some instances, intermediate or pre-frailty is defined as positive for one or two criteria. The advantage of the Fried criteria is that they provides a specific definition of frailty, which provides a method to compare populations from different studies. Furthermore, the Fried criteria use data from multiple aspects, capturing the multiple domains of frailty. The disadvantage of this method is that the dichotomous classification may not capture mild frailty. Also, the measurements are a mix of objective and self-reported which could be difficult to standardize.

The Frailty Index was established by Rockwood et al. in 2006 and provides a continuous definition of frailty. In this methodology, clinical problems are recorded as present or not present in a patient, obtained using a pre-determined list. The number of present deficits is divided by the total number of clinical problems on the pre-determined list to give a proportion. With this method more deficits indicates a higher degree of frailty. The advantage of the Frailty Index is its flexibility. Since there is no cutoff point for frailty, this method defines participants as plots on a continuum rather than grouping them as frail or non-frail. However, it can be difficult to standardize if different criteria are used for the diagnosis of an individual deficit. Furthermore, the measurements can also be a mix of objective and self-reported measures.

The Short Physical Performance Battery (SPPB) was developed in 1994 by Guralnik et al. and categorizes physical function as poor or high. For this method, gait speed, balance and chair stand times are measured and compared against standardized values, which yield a score from zero to 4 for each component. A cumulative score is then counted, providing a number from zero to twelve. The advantage of the SPPB is that it provides a robust, simple and objective method of assessing physical function that can be standardized and allow for comparison to other populations. However, this method does not capture all domains of frailty and may be less sensitive.

Wewill also collect the following data from the physician and nurse who have seen the participant during their clinic visit. 1. ‘Do you think the patient is frail?’, 2. ‘Rate the patient’s level of frailty (1 = very fit, 5 = very frail), 3. ‘If the patient chooses dialysis, do you think they will live > 6 months?’, and 4. ‘If the patient chooses dialysis, do you think they will have a “Good” quality of life?’. If the participant was already on dialysis at the time of the visit, Question 3 and 4 are omitted. While this method is subjective, we are interested in whether the perception of the health care providers has correlation with other frailty measures.

At the 3 year mark of the study, the study database will be linked to databases (see *Study Outcomes*) using Personal Health Information Numbers (PHIN), allowing information about hospitalizations and health outcomes to be collected. Laboratory Samples will be analyzed for biomarkers, such as cystatin C and FGF-23 at a later date.

Concurrently with this prospective cohort study, we are conducting systematic reviews and pilot clinical trials of several nutrition and exercise interventions for reducing frailty in patients with CKD. We hope to enroll study participants in randomized trials for the most promising interventions after 3 years of observation.

### Analysis

#### Preliminary analysis

The preliminary data presented here include data on SPPB measurements for the first 217 participants. For the preliminary analysis, participants were grouped by SPPB scores, score ≥10 (normal physical function) and score <10 (reduced lower extremity physical function). For the purposes of this report, we have defined frailty using physical function criteria only. In future reports, we aim to utilize a multi-dimensional definition of frailty.

Continuous variables are expressed as median (interquartile range) and compared using Mann–Whitney Test; categorical variables are expressed as n (%) and compared using Chi-Square or Fisher Exact Test. For the preliminary analysis, a logistic regression model was created using the baseline data (participants who had undergone at least one assessment). The primary outcome of the model was SPPB score <10, which is defined as frail (reduced physical function). In the preliminary analysis, we examined the association between age, gender and common comorbid conditions in patients with CKD with a frailty definition based on the SPPB.

## Results

### Baseline characteristics

The baseline characteristics of the CanFIT cohort can be found in Table 2. To date, 217 participants completed a baseline assessment and 52 participants have completed their first follow-up assessment. An additional 137 potential participants were approached but not suitable for inclusion to the study. Of these, 116 declined to participate, 9 were unable to speak English, 7 had severe visual or hearing impairment and 5 had known cognitive impairment. Data were collected from 2 of the 4 sites (Seven Oaks General Hospital and St. Boniface General Hospital) as these were the first sites to begin enrolment.

**Table 2:** Baseline characteristics of the study population to date

Variable	Full Cohort (N = 217)	SPPB ≥ 10 (N = 95)	SPPB < 10 (N = 122)
Age (years)	70.3 (60–79.1)	62.9 (52.9–72.3)	74.6 (63.4–82.5)
Gender (Female)	84 (40.0 %)	27 (29.4 %)	57 (48.3 %)
Weight (kg)	83 (71.7–96.6)	81.2 (72–95.9)	86.1 (71.7–96.6)
Systolic Blood Pressure (mmHg)	137 (124–151)	135 (123–149)	140 (126–151)
Diastolic Blood Pressure (mmHg)	74 (66–82)	78 (68–85)	71 (65–78)
Pulse Pressure (mmHg)	64 (52–76)	59 (48–70)	69.5 (57–79)
Laboratory Values			
eGFR (mL/min/1.73 m^2^)	19 (14–27)	18 (13–26)	20 (16–27)
Creatinine (umol/L)	250.5 (189–334)	273.5 (202–404)	229 (186–309)
Log Urine ACR	3.3 (1–5.1)	3.3 (1.1–5)	3.3 (0.9–5.2)
Hemoglobin (g/L)	114 (107–124.5)	118 (110–126)	113 (105–122)
Hemoglobin A1c (%)	6.3 (5.7–7.9)	5.9 (5.5–7.5)	6.8 (5.8–8.1)
Blood Glucose (mmol/L)	6.6 (5.4–9.6)	6.2 (5.3–8.5)	7.3 (5.5–9.9)
Comorbidities			
Visual or Hearing Impairment	101 (46.5 %)	32 (33.7 %)	69 (56.6 %)
Hypertension	181 (85.0 %)	76 (80.0 %)	105 (89.0 %)
Dyslipidemia	133 (63.0 %)	55 (57.9 %)	78 (67.2 %)
Diabetes (Type I or II)	128 (60.4 %)	40 (42.1 %)	88 (75.2 %)
Previous MI	29 (13.7 %)	8 (8.4 %)	24 (20.5 %)
Congestive Heart Failure	31 (14.6 %)	5 (5.3 %)	24 (20.5 %)
Neurologic Disease	51 (24.1 %)	3 (3.2 %)	9 (7.7 %)

Note: Continuous variables are expressed as Median (Interquartile Range) and compared using Mann–Whitney Test; Categorical variables are expressed as N(%) and compared using Chi-Square or Fisher Exact Test

Neurologic Disease contains stroke, transient ischemic attack and Parkinson’s disease. *ACR* Albumin Creatinine Ratio, *MI* Myocardial Infarction

### Short physical performance battery

The SPPB data from 217 participants were analysed and are reported in Table 3. 122 (56 %) participants were defined as reduced physical function (SPPB score <10) and 95 participants were defined as having normal physical function (SPPB score ≥10). The overall mean score for SPPB was 8.04, while the mean score by section was: Chair stand: 2.02, Balance tests: 2.98 and 4 m gait speed test: 3.10.

**Table 3:** Baseline characteristics of patients stratified by SPPB

Variable	SPPB ≥ 10 (N = 95)	SPPB < 10 (N = 122)	*P*-Value
Age (years)	62.9 (52.9–72.3)	74.6 (63.4–82.5)	<0.0001
Gender (Female)	27 (29.4 %)	57 (48.3 %)	0.0054
Weight (kg)	81.2 (72–95.9)	86.1 (71.7–96.6)	0.3352
Systolic Blood Pressure (mmHg)	135 (123–149)	140 (126–151)	0.2047
Diastolic Blood Pressure (mmHg)	78 (68–85)	71 (65–78)	0.0008
Pulse Pressure (mmHg)	59 (48–70)	69.5 (57–79)	<0.0001
Laboratory Values			
eGFR (mL/min/1.73 m^2^)	18 (13–26)	20 (16–27)	0.0901
Creatinine (umol/L)	273.5 (202–404)	229 (186–309)	0.0273
Log Urine ACR	3.3 (1.1–5)	3.3 (0.9–5.2)	0.7659
Hemoglobin (g/L)	118 (110–126)	113 (105–122)	0.0107
Hemoglobin A1c (%)	5.9 (5.5–7.5)	6.8 (5.8–8.1)	0.0014
Blood Glucose (mmol/L)	6.2 (5.3–8.5)	7.3 (5.5–9.9)	0.0793
Comorbidities			
Visual / Hearing Impairment	32 (33.7 %)	69 (56.6 %)	0.0008
Hypertension	76 (80.0 %)	105 (89.0 %)	0.0682
Dyslipidemia	55 (57.9 %)	78 (67.2 %)	0.1617
Diabetes (Type I or II)	40 (42.1 %)	88 (75.2 %)	<0.0001
Previous MI	5 (5.3 %)	24 (20.5 %)	0.0013
Congestive Heart Failure	5 (5.3 %)	26 (22.2 %)	0.0005
Neurologic Disease	12 (12.6 %)	39 (33.3 %)	0.0005

Note: Continuous variables are expressed as Median (Interquartile Range) and compared using Mann–Whitney Test; Categorical variables are expressed as N(%) and compared using Chi-Square or Fisher Exact Test

Neurologic Disease = stroke, transient ischemic attack, Parkinson’s disease, *ACR* Albumin Creatinine Ratio, *MI* Myocardial Infarction

### Factors associated with reduced physical function (SPPB < 10)

Older age, higher weight and higher pulse pressure were associated with reduced physical function. Diabetes mellitus (Type 1 or 2), dyslipidemia and hypertension were more prevalent in the reduced physical function group. Participants in the frail group were also more likely to have suffered a cardiac or vascular event (16.8 % vs 41.9 %). In our regression model, older age, female gender and presence of diabetes were associated with frailty. Cardiovascular and peripheral vascular disease were not associated, and the presence of congestive heart failure had a trend towards a positive association. (OR 2.74 95 % CI0.86–8.75) (Table 4).

**Table 4:** Factors associated with SPPB Score <10

Model 2			
Variable	OR	95 % CI	*P*-Value
Age (years)	1.09	1.05–1.12	<0.0001
Gender (Female)	3.50	1.69–7.22	0.0007
Diabetes (Type I or II)	6.95	3.18–15.20	<0.0001
Cardiac Issue	1.25	0.52–3.01	0.6200
Peripheral vascular disease	1.14	0.40–3.23	0.8052
Congestive heart disease	2.74	0.86–8.75	0.0900
Area under the ROC curve: 0.831 (0.776–0.885)			

All Variables in model 5 were considered in stepwise selection. Cardiac Issue = MI, Previous Angioplasty or Stent, Previous Cardiac Surgery. *ACR* Albumin Creatinine Ratio, *CI* Confidence Interval, *OR* Odds Ratio, *MI* Myocardial Infarction, *TIA* Transient Ischemic Attack

## Discussion

In the 217 people recruited to date in the CanFIT Study, 56 % of participants had reduced physical function at baseline. Participants with reduced physical function were more likely to be older, female, have a wider pulse pressure and suffer from comorbidities such as diabetes, heart disease and neurological disease. It is striking that after adjusting for age, gender and comorbidities, the risk of having reduced physical function was 7 fold higher in those with diabetes. This suggests that diabetes may lead to impairment of physical ability in patients with CKD independent of overt changes in kidney function or cardio/cerebrovascular events. Future analyses from CANFIT will allow us to examine other definitions of frailty, and associate them with long term outcomes.

Previous studies of frailty in CKD have primarily focused on earlier stages of CKD, or on patients receiving dialysis, and have used the Fried Criteria to define frailty. Our study will define frailty using multiple definitions and has already identified a high burden of frailty at time of study enrollment using the SPPB. Interestingly, eGFR is not associated with reduced physical function in our study cohort, unlike the results of some other recent studies [15, 16]. This may be a result of the narrow selection of kidney function of our population (eGFR < 30 mL/min/1.73 m^2^) and the potential limitations of using serum creatinine based equations for determining kidney function.

Previous studies have also examined the association between frailty and adverse outcomes in patients with CKD and have determined that coexistence of CKD and frailty leads to poorer outcomes, such as death or dialysis [9, 15]. These studies however have been in patients with earlier stages of CKD, and have had limited ability to determine the association between CKD, frailty and dialysis modality decisions. Similarly, the lack of repeated measures of physical and cognitive function in the existing literature has also limited the understanding of the natural history of the CKD-Frailty interaction.

### Strengths

The CanFIT Study represents a novel opportunity to observe the clinical history of people with CKD and the co-occurrence of frailty and comorbid conditions. We hope that longitudinal analysis of our cohort will provide valuable insight into the links between frailty and outcomes in CKD. Our study is unique because we plan to assess frailty longitudinally in people with advanced CKD, following participants as they transition to different dialysis modalities or to conservative (non-dialysis) care. Our inclusion of people who are not frail will allow us to describe the development of new-onset frailty, and identify its predictors. In the short term, our goal is describe the trajectory of frailty and its relationship with other patient characteristics. The diversity of data collected will allow us to define frailty in our population using multiple constructs. Conducting the study in a single-payer system will also allow us to minimize attrition over long term follow up. In the long term, we aim to understand more about the pathophysiology of frailty in CKD patients and to investigate the feasibility and effectiveness of targeted interventions to improve their physical and cognitive function and eventually improve patient outcomes. These interventions may range from nutritional and physical activity based treatments that target physical function to care pathways that better accommodate the needs of the frail patient. We are presently conducting scoping and systematic reviews to identify deficits in the literature and hope to begin prospective studies in the upcoming years.

### Limitations

There are some limitations to our study. First, all of our patients have an eGFR < 30 ml/min/1.73 m^2^ and are referred to be cared for by specialized interprofessional CKD clinic teams. As such, our findings may not be generalizable to patients with earlier stages of CKD, unreferred populations or those on dialysis. Our target population was chosen as it was underrepresented in previous studies of frailty and CKD, and is likely to have a high incidence of adverse outcomes (particularly dialysis starts) given the lower level of kidney function. Secondly, some of the measures in our study, such as physical activity and cognitive function, are determined using validated questionnaires. These instruments are less accurate than gold standard tests such as accelerometry or a detailed neurocognitive battery, but were chosen for ease of administration by decreasing responder burden. In the future, we may conduct sub studies incorporating more detailed measures in a subset of the original study population. Third, we decided not to use substitute decision maker’s consent to enrol patients who were unable to provide informed consent because of dementia or language barriers: this may introduce bias by excluding those with the most severe cognitive impairment and it reduces our ability to include all ethnic and cultural groups. Omission of these patients could affect the description of burden of frailty in this population, however, the number of patients excluded to date for this reason was small (2 patients). In our future analyses, we will analyse the physician and nurse impression of frailty, which are variables that have not been validated, to our knowledge. Finally, our study uses multiple research coordinators to collect data, which could introduce inter-operator variability. We will minimize this through detailed standard operating procedures, extensive standardized training, and ongoing data audits. We will use statistical methods to identify differences between study sites and research coordinators and assess for inter-operator variability.

### Summary

The preliminary results of the CanFIT Study demonstrate the feasibility of our study. We have also identified a strong association between diabetes and frailty in people with CKD. In future analyses, the CanFIT Study cohort will provide further insight into the clinical history of frailty and advanced CKD and its impact on dialysis decisions, treatment and outcomes with a view to investigate the feasibility and effectiveness of targeted interventions to improve outcomes.
